# Large-Scale Functional Analysis of CRP-Mediated Feed-Forward Loops

**DOI:** 10.3390/ijms19082335

**Published:** 2018-08-09

**Authors:** Chi-Dung Yang, Hsi-Yuan Huang, Sirjana Shrestha, Yen-Hua Chen, Hsien-Da Huang, Ching-Ping Tseng

**Affiliations:** 1 Institute of Bioinformatics and Systems Biology, National Chiao Tung University, Hsinchu 300, Taiwan; chidung1212@gmail.com (C.-D.Y.); aliken.bi93g@nctu.edu.tw (H.-Y.H.); sirju10@yahoo.co.in (S.S.); 2 Institute of Population Health Sciences, National Health Research Institutes, Miaoli 350, Taiwan; 3 Department of Biological Science and Technology, National Chiao Tung University, Hsinchu 300, Taiwan; 4 Warshel Institute for Computational Biology, The Chinese University of Hong Kong (Shenzhen), Shenzhen 518172, China; 5 School of Science and Engineering, The Chinese University of Hong Kong (Shenzhen), Shenzhen 518172, China; 6 Department of Laboratory Medicine, China Medical University Hospital, Taichung 420, Taiwan; 7 Department of Microbiology and Immunology, Weill Cornell Medicine, Cornell University, New York, NY 10021, USA; w0a0csnark@gmail.com

**Keywords:** feed-forward loop (FFL), cAMP receptor protein (CRP), transcription factor (TF)

## Abstract

Feed-forward loops (FFLs) represent an important and basic network motif to understand specific biological functions. Cyclic-AMP (cAMP) receptor protein (CRP), a transcription factor (TF), mediates catabolite repression and regulates more than 400 genes in response to changes in intracellular concentrations of cAMP in *Escherichia coli*. CRP participates in some FFLs, such as *araBAD* and *araFGH* operons and adapts to fluctuating environmental nutrients, thereby enhancing the survivability of *E. coli*. Although computational simulations have been conducted to explore the potential functionality of FFLs, a comprehensive study on the functions of all structural types on the basis of in vivo data is lacking. Moreover, the regulatory role of CRP-mediated FFLs (CRP-FFLs) remains obscure. We identified 393 CRP-FFLs in *E. coli* using EcoCyc and RegulonDB*.* Dose–response genomic microarray of *E. coli* revealed dynamic gene expression of each target gene of CRP-FFLs in response to a range of cAMP dosages. All eight types of FFLs were present in CRP regulon with various expression patterns of each CRP-FFL, which were further divided into five functional groups. The microarray and reported regulatory relationships identified 202 CRP-FFLs that were directly regulated by CRP in these eight types of FFLs. Interestingly, 34% (147/432) of genes were directly regulated by CRP and CRP-regulated TFs, which indicates that these CRP-regulated genes were also regulated by other CRP-regulated TFs responding to environmental signals through CRP-FFLs. Furthermore, we applied gene ontology annotation to reveal the biological functions of CRP-FFLs.

## 1. Introduction

Microbes must accustom to environmental changes produced by physiological signals, such as nutrients, salts and molecular species and physicochemical states, such as temperature, redox potential and osmolality to survive [1]. *Escherichia coli* regulates gene expressions using considerable different gene regulators, such as transcription factors (TFs), to react to those environmental signals [2].

**Figure 1:**
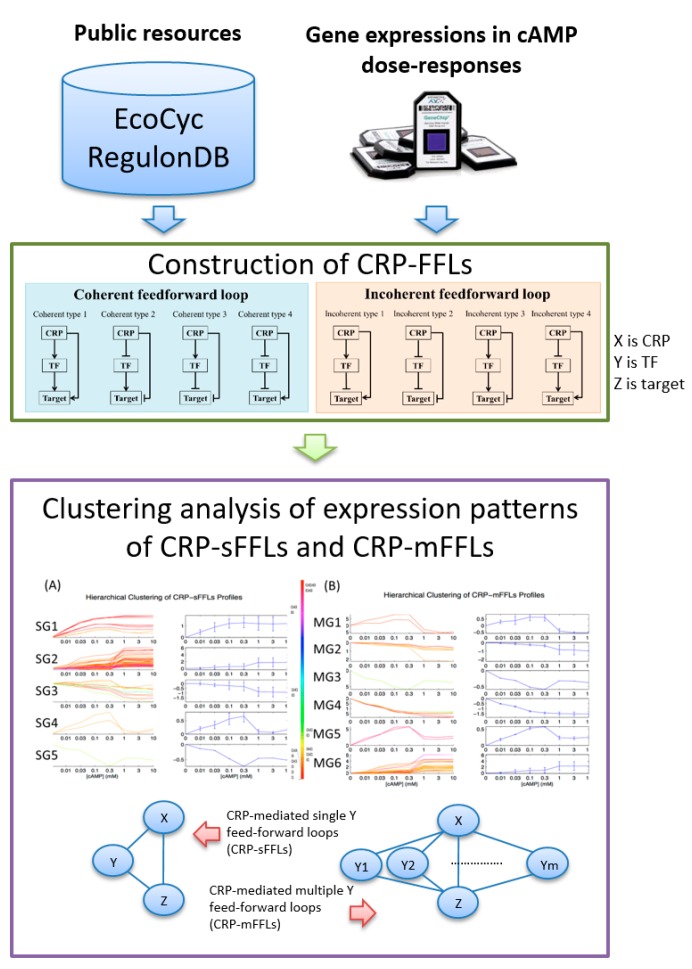
Data analysis flow of cyclic-AMP receptor protein–feed forward loops (CRP-FFLs). Data analysis proceeds in two steps: (i) utilization of public resources and gene expressions in cAMP dose–responses for construction of CRP-FFLs (adapted from reference [11]) and (ii) hierarchical clustering analysis of gene expressions to reveal hidden patterns in both CRP-sFFLs and CRP-mFFLs.

Databases such as EcoCyc 22.0 [3] and RegulonDB 9.4 [4,5] have reported 201 TFs in *E. coli*. A complicated gene regulation network (GRN) is formed by linking the regulation information between TFs and their targets. Numerous computational analyses have attempted to assess the importance of TFs, which include information on the number of TF target genes and interactions among other TFs [6,7,8]. Complex transcription regulation networks (TRN) are investigated by analyzing small blocks of networks called “network motifs”. A feed-forward loop (FFL) is an important network motif in *E. coli* gene regulation that contains two TFs (*X* and *Y*) and one or more genes. In an FFL, TF *X* regulates TF *Y. X* and *Y* regulate target gene *Z* and jointly regulates its transcription rate. Depending on the definition, eight types of FFLs are formed. An FFL is “coherent” if the direct effect (positive or negative) of *X* on *Z* is the same as the indirect effect through the second TF *Y*. On the contrary, if the direct effect of *X* on gene *Z* is opposite from the indirect effect through *Y*, the FFL is “incoherent”. The four types of coherent FFLs are coherent type 1 (Coh1), coherent type 2 (Coh2), coherent type 3 (Coh3) and coherent type 4 (Coh4) and those of incoherent FFLs are incoherent type 1 (InCoh1), incoherent type 2 (InCoh2), incoherent type 3 (InCoh3) and incoherent type 4 (InCoh4) [9,10]. The eight types of FFLs (Coh1–InCoh4), labeled as “construction of CRP-FFLs”, are illustrated in Figure 1. In *E. coli*, coherent FFLs serve as a sign-sensitive delay element in an *ara* system [11] and incoherent FFLs accelerate the response time of a gal system [12]. In addition, two other simple topological generalizations of FFL, which are called multi-Y and multi-output FFLs, are obtained by replicating the appropriate nodes [13]. In the case of multi-Y FFLs, the target gene *Z* is regulated by single TF *X* and multiple *Y*. In the case of multi-output FFLs, the multiple target genes *Z* are simultaneously regulated by a single TF *X* and *Y*.

The basic theory of FFL has been applied in different fields. For example, FFL can be easily adjusted to produce a single-pulse response and further regulate blood sugar in diabetic patients [14]. In human disease, the latest research has found that the interaction of FFL members, miRNA, TF and gene interaction may promote effective treatment of ischemic stroke [15]. FFL also plays an important role in maintaining homeostasis in biological response [16]. However, in microbiology, although biological databases have documented the regulatory relationship between TFs and genes, integrated experiments and analysis are insufficient. For the first time, we have presented a comprehensive FFL study. We chose *E. coli* as a research model and achieved a complete analysis of CRP-mediated FFLs.

In *E. coli*, CRP has been reported to be a global TF that regulates over 400 genes [17,18]. In CRP-mediated FFLs (CRP-FFLs), CRP and a CRP-regulated TF are denoted as *X* and *Y*, respectively. Although Alon et al. reported the kinetic features of CRP-FFLs in *E. coli* [12], only Coh1, Coh2 and Coh4 FFLs were reported due to the limitation of a small sample size to support statistical significance and information on gene interaction. Evidence indicated that substantial potential FFLs exist in the CRP regulon but data on the interaction between gene regulations do not provide a thorough understanding of the biological functions of CRP-FFLs in *E. coli.* In addition, databases such as EcoCyc and RegulonDB have revealed that various genes are involved in multi-Y FFLs such as GadA, a glutamate decarboxylase enzyme coded by the *gadA* gene, which confers resistance to acid conditions and is regulated by CRP and CRP-regulated TFs, GadX and FIS. With CRP, GadX and FIS can co-regulate *gadA* gene expression and form Coh2 and InCoh2 FFLs. In the following section, we describe single-Y and multi-Y FFLs involved in CRP-FFLs as CRP-mediated single-Y FFL (CRP-sFFL) and CRP-mediated multi-Y FFL (CRP-mFFL), respectively (Figure 1). We define a CRP-sFFL as an FFL that contains CRP and one CRP-regulated TF and a CRP-mFFL as a motif that involves CRP and two or more CRP-regulated TFs to regulate one target gene.

To clarify the functional CRP-FFLs in *E. coli*, cAMP dose–response analysis was performed by microarray, thus determining the capacity of CRP to regulate target gene expression involved in CRP-FFLs at various concentrations (0–10 mM) of cAMP. Accurately controlling the activity of CRP in cAMP dose–response experiments requires maintaining intracellular concentration of cAMP. Therefore, we applied the *cyaA* and *tolC* double mutant strain that has lost the ability to produce and exhaust cAMP to measure the relative expression level of a target gene in the presence or absence of cAMP [19].

This study aims to establish a comprehensive understanding of the biological functions of CRP-FFLs by combining known gene regulation information and dose–response microarray experiments. The CRP-FFLs can be further divided into CRP-sFFLs and CRP-mFFLs, which exist in various expression patterns. We combined target genes with similar profiles for gene ontology (GO) enrichment analysis to further explore the functional categories of these genes. In brief, we provided the first comprehensive study using CRP-FFLs to understand how *E. coli* adapts to environmental changes under various concentrations of cAMP (Figure 1).

## 2. Results

### 2.1. CRP-Mediated FFLs in E. coli

**Figure 2:**
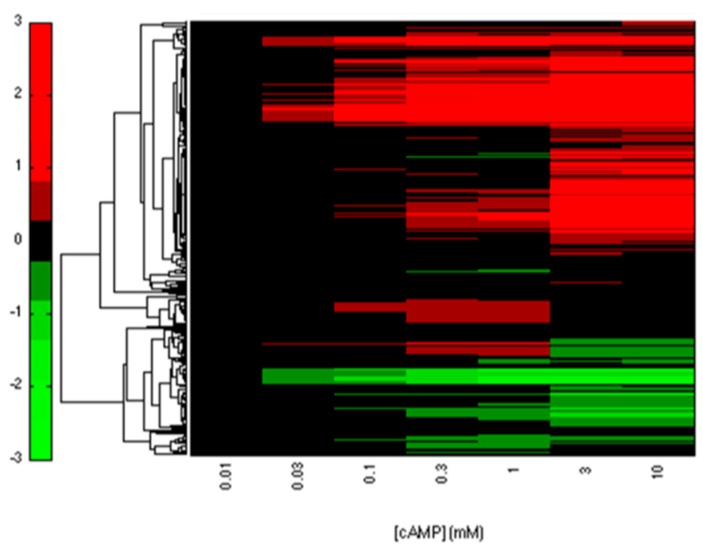
Heatmap of CRP-regulated genes. The clustering microarray data of the reported CRP-regulated genes are summarized in the heatmap. The expression profile of genes was used to construct CRP-FFLs for further analysis.

**Figure 3:**
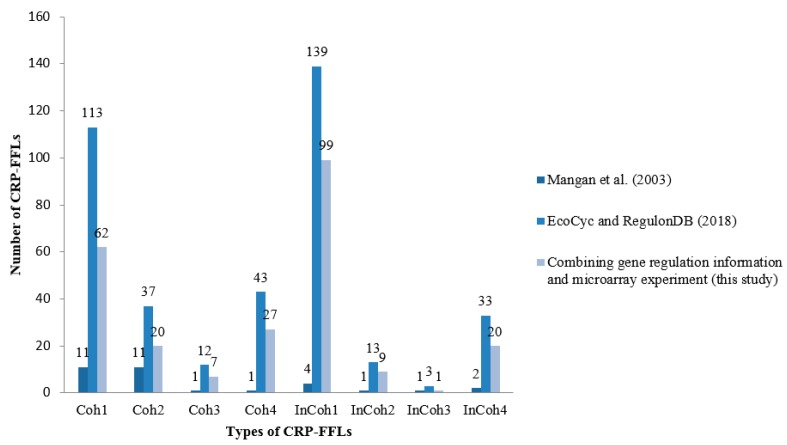
Distribution of CRP-FFLs in *E. coli*. Regulation information was based on the statistics obtained from Mangan et al. (2003) [10], EcoCyc and RegulonDB and CRP-FFLs generated in this study. Some CRP-regulated genes reported in the literature and public databases did not show significant differential expression in our experimental conditions, probably because these genes require other TFs that are only expressed in specific conditions for a cooperative response to cAMP.

We collected 3958 interactions between TFs and genes from EcoCyc and RegulonDB. Among these interactions, CRP regulates 432 genes that contain 51 TFs. From these genes, we identified 393 CRP-FFLs consisting of 292 genes and 43 TFs. To obtain and clarify the functional CRP-FFLs in *E. coli*, cAMP dose–response experiments were performed by microarray as follows. In this study, all of the microarray data were shown as fold-change value relative to zero cAMP control. We first filtered out the genes with relative expression levels of all cAMP added samples relative to zero cAMP control that are less than the 0.5 log2 fold-change value from microarray data. Microarray results are summarized in a heat map in Figure 2 and Supplementary Materials File S1. Finally, we obtained 202 CRP-FFLs consisting of 147 genes and 37 TFs to determine the optimal number of clusters for each type of CRP-FFL. The statistics of data in eight types of CRP-FFLs is shown in Table 1. The distribution of CRP-FFLs in *E. coli*, based on the statistics of Mangan et al. [10], EcoCyc and RegulonDB and CRP-FFLs in this study, is compared in Figure 3 and Supplementary Materials File S1. Some previously reported CRP-regulated genes did not exhibit significant differential expressions in our experimental conditions in Supplementary Materials File S2, probably because these genes require other TFs expressed in some specific environmental conditions [17,18]. The data were used to construct CRP-FFLs, which were then subjected to further analysis.

### 2.2. CRP-FFLs Characterized by Distinct cAMP Dose–Responses

**Figure 4:**
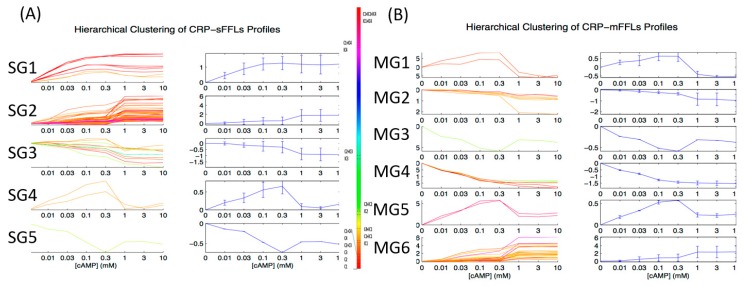
Expression profiles of CRP-sFFLs and CRP-mFFLs. Colored CRP-FFLs are used to distinguish between the various CRP-FFL types. The CRP-FFLs were divided into five groups (SG1 to SG5) in CRP-sFFLs and six groups (MG1 to MG6) in CRP-mFFLs according to distinct cAMP dose–response profiles. Hierarchical clustering results for (**A**) CRP-sFFLs and (**B**) CRP-mFFLs.

After the CRP-FFLs were found, we subjected the target genes of all available CRP-FFLs to hierarchical clustering on the basis of the cAMP dose–response microarray data. We divided CRP-FFLs into two groups, CRP-sFFLs and CRP-mFFLs, to investigate the relationship between them. Records from EcoCyc and RegulonDB along with the microarray data were used to distinguish 101 CRP-sFFLs and 101 CRP-mFFLs among 202 CRP-FFLs. Figure 4A presents the expression profile of target genes regulated by CRP-sFFLs, whereas Figure 4B presents the CRP-mFFL profile of target genes regulated by more than one CRP-regulated TF. We investigated whether each of the CRP-FFLs could be assigned to a specific cluster. Furthermore, according to their distinct cAMP dose–response profiles, the CRP-FFLs were divided into five groups (from SG1 to SG5) in CRP-sFFLs and six groups (from MG1 to MG6) in CRP-mFFLs. 

For example, both groups SG2 in CRP-sFFLs and MG6 in CRP-mFFLs can form similar expression profiles as cAMP increases (Figure 4A,B). The cAMP dose–responses are nearly identical and characterized by significant levels of CRP-FFLs until cAMP reaches a high concentration. These results indicate that the dose–response of each type of CRP-FFL may be related to their topology and transcriptional regulation with CRP and CRP-regulated TFs.

### 2.3. GO Enrichment Analysis of FFLs in CRP Regulatory Network

Considering that CRP-sFFLs and CRP-mFFLs share similar cAMP dose–response profiles, we combined the genes of similar profiles for GO enrichment analysis to further explore the functional categories of these genes. The combinations of CRP-sFFL groups and CRP-mFFL groups are shown in Table 2 and Supplementary Materials File S2. According to the results of GO enrichment analysis, we classified the target genes of CRP-sFFLs and CRP-mFFLs into five functional groups (FG1 to FG5).

Carbohydrates are the main carbon source for ATP synthesis and they also serve as signals from the environment. CRP is a well-known global regulator of carbohydrate metabolism [18]. We surveyed the FFLs involved in functions related to sugar metabolism to determine which CRP-FFLs were used by *E. coli* for various carbon sources. Most of the CRP-mediated Coh1 and InCoh1 FFLs were involved in the regulation of sugars. Mangan et al. [11] mentioned that cAMP is produced when the concentration of glucose was low in the cell; therefore, we expected that the concentration of cAMP would be sufficiently high to form the cAMP–CRP complex and regulate CRP-regulon in intestinal bacteria. In agreement with the findings of Alon [12], our cAMP dose–response data indicated that the gene expression of most target genes involved in Coh1 and InCoh1 FFLs in carbon utilization pathways increased significantly under high concentrations of cAMP. The results also showed that groups SG2 and MG6 were dominated by CRP-FFLs with the transporter function and carbon source utilization. By contrast, the CRP-FFLs involved in the iron and pH homeostasis preferred to use all CRP-FFL types except the Coh3 FFL. Other features are summarized in Table 3 and Supplementary Materials File S2. Many CRP-FFLs were involved in more than one functional group. Thus, a further study on the biological functions of specific FFLs in different biological activities is beneficial.

## 3. Discussion

### 3.1. Integration of Different FFL Types Diversifies the Output Response

One of the key questions regarding the function of network motifs is whether a simple three-gene FFL is sufficient and/or necessary to perform a specific biological function. Moreover, most current studies in bioinformatics depend solely on preexisting gene integration data and are thus limited by the number of FFLs that have been identified [20]. In this study, we focused on a master TF to improve the CRP regulon by combining cAMP dose–response microarray data.

Although the proposed approach has enriched our knowledge of the structure–function relationships in CRP-mediated gene interaction networks, uncertainties occur due to several factors, such as the presence of unknown transcriptional regulations and a TF that sometimes serves as a dual regulator [2]. Therefore, a structure, such as the described CRP-sFFL, may indeed include more complex regulatory networks, such as CRP-mFFLs or feedback regulations. To minimize bias resulting from the lack of comprehensive gene interaction data, we compared the clustering results of the cAMP dose–response profiles for CRP-sFFLs and CRP-mFFLs and unexpectedly found the profiles to be quite similar, with the clustering result shown in Figure 4. In fact, four out of five expression profile clusters derived from CRP-sFFLs were also observed in CRP-mFFLs. In this case, two possible explanations can be provided. On the one hand, the so-called CRP-sFFLs might be indeed part of CRP-mFFLs because of the limited gene interaction data between CRP-regulated TFs and target genes. On the other hand, the profile of CRP-mFFLs might be dominated by a master CRP-sFFL whose promoter had a higher affinity or multiple CRP binding sites and thus presents a profile similar to that of a specific CRP-sFFL. If the latter turns out to be the case, we expect that the frequency of FFLs in the cAMP dose–response profile of CRP-sFFLs would be similar to that of CRP-mFFLs in a similar profile.

Table 3 provides detailed information for CRP-sFFLs and CRP-mFFLs. Based on the hierarchical clustering results, the expression profiles of the target genes in CRP-sFFLs and CRP-mFFLs can be subdivided into five and six groups, respectively. While comparing the hierarchical clustering results of CRP-sFFLs and CRP-mFFLs, we found that the group SG1 (FG1) profiles are only observed in CRP-sFFLs and this group is dominated by Coh1 and InCoh1 FFLs. By contrast, four other groups, namely, SG2 (FG1), SG3 (FG3), SG4 (FG4) and SG5 (FG5), share similar profiles with those of the CRP-mFFLs. Given that CRP acts as an activator in Coh1 and InCoh1 FFLs and the average correlation coefficient (approximately 0.99) of profiles is high in the SG1 (FG1) group, we hypothesized that this profile pattern represents one of the basic profiles of Coh1 and InCoh1 FFLs in CRP-sFFLs.

However, our results also demonstrated that one FFL could be used in more than one dose–response profile. The genes in groups SG2 (FG2) and MG6 (FG2) were all regulated by Coh1, Coh4, InCoh1 and InCoh4 FFLs regardless of whether they were regulated by CRP-sFFLs or CRP-mFFLs. All FFL combinations that gave rise to a dose–response profile similar to that of the group genes were composed of a combination of Coh1, Coh4, InCoh1 and InCoh4 FFLs. Notably, CRP acts as an activator in these FFLs, suggesting that CRP alone was sufficient to increase the expression of the target genes, whereas the transcriptional regulation of other TFs was required to modulate the expression of target genes when the cAMP concentration was above 0.3 mM.

The expression profiles in the SG3 (FG3) group can be observed in most CRP-sFFLs except Coh3 and InCoh4 FFLs. In this cluster, the expression of target genes decreases as the concentration of cAMP increases. The decrease of the target genes fits into a linear form in the log scale. In general, this group is a counterexample of the SG2 (FG3) group because CRP acts as a repressor in Coh2, InCoh2 and InCoh3 FFLs. In agreement with the aforementioned observation of combining FFLs, a composition of Coh2 with InCoh2 FFLs in the MG4 (FG3) group can produce a profile similar to that in the SG3 (FG3) group. Nevertheless, some FFLs, such as Coh1, InCoh1 and InCoh4 and their combination in the MG2 (FG3) group also form a dose–response profile similar to that in the SG3 (FG3) group. Although a CRP acting as an activator regulates these FFLs, the activity of *Y* may be higher than that of CRP. If this *Y* is an activator, when the DNA binding activity of the CRP increases along with the concentration of cAMP, the occupation of *Y* on the promoter of the target gene is gradually replaced by CRP, resulting in a decrease of *Z* gene expression. By contrast, for Coh4 and InCoh1 FFLs where *Y* is a repressor, the repression effect of *Y* may outweigh the strength of CRP.

In addition, with increasing or decreasing concentrations of cAMP, some FFLs showed a bell-shaped dose–response curve in the SG4 (FG4) group (Figure 4) and the expression of the target genes in this group peaked when the concentration of cAMP ranged from 0.1 to 0.3 mM. Theoretically, the target gene should be regulated by two arms of regulation with opposite effects to achieve a bell-shaped response curve [21]. Therefore, we can expect that some incoherent FFLs can produce such a profile. Indeed, an InCoh1 FFL in CRP-sFFLs can generate a bell-shaped profile. Interestingly, the combination of Coh2 and Coh3 FFLs in the MG1 (FG4) group or InCoh1 and InCoh4 FFLs in the MG5 (FG4) group can generate similar profiles, probably because these combinations include TFs with opposite regulation effects.

In contrast to the bell curve, some combinations of FFLs were characterized by a good curve distribution, which is an inverse bell curve. In CRP-sFFLs, InCoh2 FFLs in the SG5 (FG5) group exhibited a good curve dose–response profile similar to the combination of Coh2 with InCoh2 FFLs in the MG3 (FG5) group. Our findings demonstrated that each FFL can produce some specific dose–response profiles, which can be generated by combining specific FFLs. These results not only provide clues for the biological functions of FFLs but also highlight the principles of simplified complex gene regulatory networks with CRP-mFFLs. Table 3 provides detailed information on CRP-sFFLs and CRP-mFFLs.

### 3.2. Functional Analysis of FFLs in CRP-Regulated Networks

An analysis of cAMP dose–response profiles derived from the target genes in either CRP-sFFLs or CRP-mFFLs revealed that they share four similar response profiles. We further conducted a GO analysis to explore the biological significance of these motifs. Strikingly, genes within the same clusters were involved in the same biological pathways. Indeed, the more similar the biological processes in which the genes participated, the more similar are their dose–response profiles under various concentrations of cAMP. The GO analysis revealed five major functional groups, each involving a group of genes demonstrating similar cAMP responses and network motifs.

Contrary to the other CRP-sFFL profile clusters, the profile of the SG1 (FG1) group was only found in target genes in CRP-sFFLs, suggesting that these profiles may only reflect the function of Coh1 and InCoh1 FFLs. Therefore, we can confirm that at least for those genes in the SG1 (FG1) group, CRP-sFFL is sufficient to generate a specific profile. The GO analysis clearly showed that the SG1 (FG1) group was predominated by genes involved in the central metabolism processes, including the tricarboxylic acid cycle, cellular respiration and generation of energy by the oxidation of organic compounds. The expression of these genes increased along with the concentration of cAMP. The increased expression begins to form a trace amount of cAMP (0.01 mM) and reached a stationary phase as the concentration of cAMP reached 1 mM. This result is in accordance with a previously reported mechanism in which the expression of TCA cycle-related genes is activated as the cell consumes environmental glucose compounds to facilitate the TCA cycle [22,23,24].

The remaining CRP-sFFL profiles were also found in the dose–responses of CRP-mFFLs except for the SG1 (FG1) group. For example, target genes in the SG2 (FG2) group of CRP-sFFLs and the MG6 (FG2) group of CRP-mFFLs were both characterized by a sigmoid curve. Interestingly, their profiles represented a switch-like behavior to accelerate the expression of target genes only if the concentration of cAMP was above 0.3 mM. In other words, the regulation allows target genes to reach their maximum expression levels until the cAMP concentration exceeds a certain threshold.

This behavior is consistent with catabolite repression, a well-known mechanism in *E. coli* [25,26]. When *E. coli* is grown in a medium with mixed carbon sources, it first consumes glucose but represses the expression of the transporters of other carbon sources. The expression of the transporters for those alternative carbohydrate compounds is activated after glucose concentrations have dropped below a certain level. This mechanism is important in energy conservation.

In contrast to the aforementioned clusters, three clusters were observed in which the expression of target genes showed an inverse correlation with the concentration of cAMP. In the SG3 (FG3) group of CRP-sFFLs and the MG2 (FG3) and MG4 (FG3) groups of CRP-mFFLs, the expressions of target genes were repressed by the cAMP-CRP complex resulting in the decrease of their expressions as the cAMP level rose. These gene profiles were found to be involved in stress response, especially in iron and pH homeostasis.

Importantly, according to the database records, CRP activated the genes in the MG2 (FG3) group; thus, this group consisted of Coh1 and InCoh1 FFLs. However, the microarray data showed an opposite trend where the expression profile was inversely correlated with the concentration of cAMP. For example, the Fec operon, composed of *fecABC* genes that encode the ferric citrate transporter, was reported to be activated by the cAMP [27], whereas our results showed that the operon is indeed repressed by the cAMP. We speculated that this is due to experimental limitations in the previous studies that did not use mutant strains to allow the precise control of cAMP levels. Further experiments, such as promoter activity assays and northern blot, are required to redefine the regulatory effect of the cAMP on these genes.

**Figure 5:**
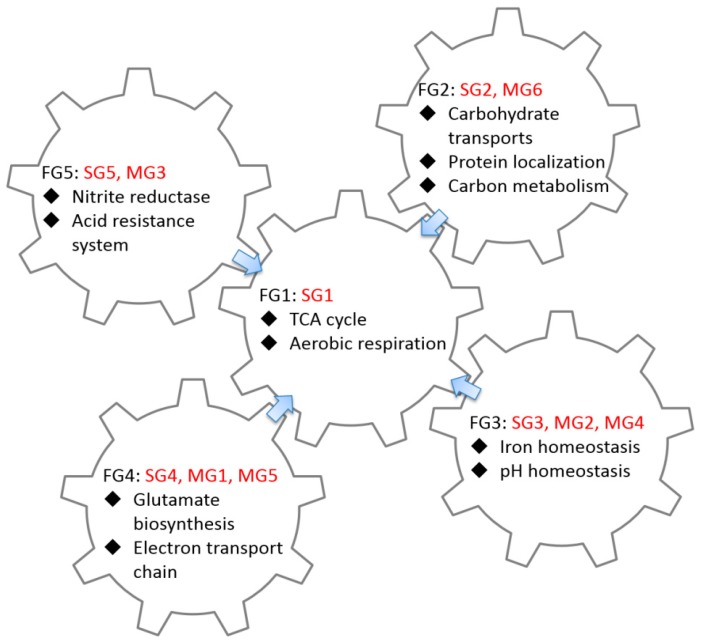
Biological interactions of CRP-FFLs. Expression patterns of target genes can be divided into five functional groups (FG1 to FG5). Each group combines single- or multi-TFs CRP-FFLs involved in different biological functions, such as carbon metabolism, transport and iron and pH homeostasis. Most functional groups in this study surround and point toward the TCA cycle.

We could not retrieve any significant GO terms due to the availability of a small number of identified genes within the SG4 (FG4) group with MG1 (FG4) and MG5 (FG4) and the SG5 (FG4) group with MG3 (FG5). The relevant biological functions and their directly associated genes are provided in Figure 5 and Table 2. The genes in the SG4 (FG4) group of CRP-sFFLs and the MG1 (FG4) and MG5 (FG4) groups of CRP-mFFLs demonstrated similar bell-shaped profiles. Interestingly, the expression levels of the MG1 (FG4) and MG5 (FG4) groups were degraded and elevated when the concentration of cAMP exceeded 1 mM compared with their basic expression levels, respectively. This condition is probably due to the high degree of intracellular cAMP activating the other unknown regulation in the MG5 (FG4) group. The genes in the FG4 functional group showed biological function in the l-glutamate biosynthetic process, electron transport chain and iron transport. One of the key processes in l-glutamate biosynthesis requires the activity of glutamate synthase, which requires NADPH. This enzyme has four substrates (l-glutamine, 2-oxoglutarate (α-ketoglutarate), NADPH and H^+^) and generates two products: l-glutamate and NADP^+^. This reaction helps bacteria produce l-glutamate using NADPH as the acceptor and iron as a cofactor. The remaining FG5 functional group was involved in the acid resistance system and nitrite reductase, as shown in Table 2.

Figure 5 illustrates the relationships between the gene expression profiles, structure of regulatory motifs and function of CRP-regulated genes. Notably, for the presented biological functions, most of the functional groups investigated in this study surrounded and pointed toward the TCA cycle. For example, the major biological function of the SG2 (FG2) and MG6 (FG2) groups is controlling the carbohydrate transporter related genes. The transport of carbon sources, especially glucose, is an important research area. When *E. coli* consumes glucose, the accumulated intracellular cAMP activates CRP, thereby enhancing the expression of glucose transport and TCA cycle-related genes. This outcome would help *E. coli* to increase glucose absorption from the environment. This combination of different CRP-FFLs allows *E. coli* to expend more glucose to promote the production efficiency of ATP in the TCA cycle. Our analysis raises applications of FFLs associated with CRP-FFLs that deserve further investigation.

## 4. Application of FFLs

The accumulation of our knowledge on each FFL contributes to an understanding of their sophisticated designs. Combining previous research on the kinetic feature of distinct FFLs [12] with our findings on the dose–response curve reveals that each FFL presents a specific control element that could potentially be applied in artificial circuit designs. Contrary to the electronic circuits, the topology and interactions between two genes can be easily changed by introducing a repressor or cooperator, or by shutting down the expression of the regulator. Thus, the expression patterns of cellular genes indicate that cells are used to cope with the current growth conditions. A more detailed investigation on how different FFLs work together will enable us to simplify the design of artificial biological circuits and define how an organism makes a specific decision in response to intracellular and environmental signals. These studies will advance a new field that uses network motifs to understand the complexity of biological functions, such as memory, asymmetric cell deviation and complex diseases.

## 5. Materials and Methods

The data analysis flow of the CRP-FFLs is shown in Figure 1. It comprises two steps: (i) construction of CRP-FFLs from public resources and gene expressions in cAMP dose–responses and (ii) hierarchical clustering analysis of gene expressions to reveal hidden patterns in both CRP-sFFLs and CRP-mFFLs.

### 5.1. Construction of CRP-FFLs

To construct CRP-FFLs, all TFs and target genes regulated by CRP were collected from two public resources, namely, EcoCyc and RegulonDB. Experimental results support the existence of regulatory interactions through both strong evidence (e.g., binding of purified proteins, DNaseI footprinting and site mutation) and weak evidence (binding of cellular extracts and transcriptional fusions). Assuming that these CRP-regulated TFs regulate the target genes, we found that some TFs and target genes that could not form CRP-FFLs were removed. In defining the eight types of CRP-FFLs, the dual or unknown regulatory effect of the TF on the regulated gene was not considered. Finally, CRP-FFLs using cAMP dose–response microarray were identified and discussed.

### 5.2. Identification of CRP-Regulated Genes Using cAMP Dose–Response Microarray

All strains used in this study were *E. coli* K12 BW25113 derivatives generated from the Keio collection system, which was provided by the National Institute of Genetics of Japan [28]. For cAMP dose–response experiments, cells were grown in a minimum medium supplied with 0.4% glucose until the log phase and then spun down and suspended in a fresh minimum medium with 0.4% glycerol and different concentrations of cAMP (0, 0.01, 0.03, 0.1, 0.3, 1, 3 and 10 mM) [19]. Cells were incubated with the cAMP medium for 15 min and then harvested for RNA isolation. To examine expression profiles of target genes involved in CRP-FFLs, the transcriptome expression of cells incubated with different cAMP concentrations using the GeneChip *E. coli* Genome 2.0 Array was compared, enabling examination of gene expression profiles of *E. coli* under various conditions to better understand the biological pathways involved. Differences in microarray expression revealed a substantial number of genes under the control of cAMP during the exponential phase.

### 5.3. Analysis of Microarray Data

Microarray experiments were performed in duplicate for each cAMP dosage. Bioconductor software written in the R statistical programming language (http://www.bioconductor.org) was used to analyze the gene expression microarray data. The microarray CEL files were background corrected and normalized and the expression value was calculated using the robust multi-chip average algorithm [29], resulting in log2 expression values. Each average expression value of treatments in the log2 ratio was compared with the cells grown in the minimum medium without cAMP.

### 5.4. Hierarchical Clustering and Functional Analysis

The hierarchical clustering analysis in the target gene expressions of CRP-FFLs from the cAMP dose–response microarray data was adopted. The insignificantly expressed genes with an absolute log2 fold change below 0.5 compared with no cAMP control were filtered out. Hierarchical clustering analysis on the basis of the Pearson’s correlation coefficient for similarity measurements and the average-linkage method was performed to group the genes affected by the metabolic change. Finally, interesting genes were classified into five functional groups using the term enrichment tool [16,30]. The maximum *p*-value 0.01 is considered to determine whether any GO terms annotate a specified list of genes at a frequency greater than what would be expected by chance.

## Figures and Tables

**Table 1:** Data statistics for each type of CRP-FFL. The detailed numbers of CRP-FFLs, transcription factors (TFs) and genes are listed for each type of CRP-FFL. A number of target genes regulated by multiple TFs and TF-regulated multiple genes are also listed. Target genes for each type of CRP-FFL are listed for further analysis.

Type	Number of CRP-FFLs	Number of TF	Number of Gene	Genes
Coh1	49	13	49	*araF*, *araG*, *dctA*, *fecA*, *fecB*, *fecC*, *fecD*, *fecE*, *fucA*, *fucI*, *fucK*, *fucO*, *fucP*, *fucR*, *fumC*, *hyfH*, *idnD*, *idnK*, *idnO*, *lamB*, *malE*, *malF*, *malG*, *malK*, *malM*, *malS*, *marB*, *ompF*, *prpB*, *prpC*, *prpD*, *prpE*, *rhaR*, *sdhA*, *sdhB*, *sdhC*, *sdhD*, *srlA*, *srlB*, *srlD*, *srlE*, *sucA*, *sucB*, *sucC*, *sucD*, *xylA*, *xylF*, *xylG*, *xylH*
Coh2	15	3	10	*gadA*, *gadB*, *gadB*, *gadC*, *gadC*, *gadE*, *gadE*, *gadX*, *gltD*, *gltF*, *mdtE*, *mdtE*, *mdtF*, *mdtF*, *proP*
Coh3	2	1	2	*gltD*, *gltF*
Coh4	26	3	26	*acs*, *actP*, *aldB*, *fadL*, *flhC*, *flhD*, *glcC*, *guaA*, *guaB*, *hlyE*, *hupB*, *mglA*, *mglC*, *mtlA*, *mtlD*, *mtlR*, *nanA*, *nanE*, *nanK*, *nanT*, *nmpC*, *xylF*, *xylG*, *xylH*, *yiaK*, *yjcH*
InCoh1	87	22	73	*cdd*, *chbB*, *cirA*, *cyoD*, *cyoD*, *cyoE*, *cyoE*, *cytR*, *entD*, *entH*, *fecA*, *fecB*, *fecC*, *fecD*, *fecE*, *fepA*, *fiu*, *flhC*, *flhD*, *fumC*, *galK*, *galK*, *galP*, *galP*, *galS*, *glcC*, *glpD*, *glpF*, *glpK*, *glpT*, *gntK*, *gntP*, *gntP*, *gntU*, *grcA*, *grcA*, *lsrA*, *lsrB*, *lsrC*, *lsrD*, *lsrF*, *lsrG*, *lsrK*, *lsrR*, *malI*, *malX*, *manX*, *manY*, *manZ*, *marB*, *mglA*, *mglA*, *mglA*, *mglB*, *mglB*, *mglB*, *mglC*, *mglC*, *mglC*, *mtlA*, *mtlD*, *mtlR*, *nagB*, *nagE*, *nupC*, *nupG*, *prpR*, *rbsA*, *rbsB*, *rbsC*, *rbsD*, *rbsK*, *rbsR*, *srlA*, *srlB*, *srlD*, *srlE*, *tsx*, *udp*, *uidA*, *uidB*, *uidC*, *uxuA*, *uxuA*, *uxuB*, *uxuB*, *xylA*
InCoh2	8	3	8	*bhsA*, *gadA*, *gadB*, *gadC*, *gadX*, *nirB*, *osmY*, *yiaJ*
InCoh3	1	1	1	*araJ*
InCoh4	14	4	14	*csgD*, *csgE*, *csgF*, *csgG*, *cyoD*, *cyoE*, *exuT*, *glpB*, *grcA*, *malE*, *malF*, *malG*, *marB*, *nrdB*

**Table 2:** Biological process ontology of CRP-FFLs. Associated GO terms and biological functions for each functional group are listed. Most functional groups with a small *p*-value are statistically significant.

Functional Group ID	Group ID	GO Term	*p*-Value	Number of Gene	Summary of Function
FG1	SG1	GO:0006099 tricarboxylic acid cycle	5.02 × 10^−12^	8	TCA cycle Aerobic respiration
		GO:0009060 aerobic respiration	8.00 × 10^−11^	8	
		GO:0045333 cellular respiration	3.13 × 10^−9^	8	
		GO:0015980 energy derivation by oxidation of organic compounds	2.18 × 10^−8^	8	
		GO:0006091 generation of precursor metabolites and energy	2.25 × 10^−7^	8	
		GO:0055114 oxidation-reduction process	7.12 × 10^−3^	8	
FG2	SG2 MG6	GO:0008643 carbohydrate transport	3.84 × 10^−26^	37	Carbohydrate transports Protein localization Carbon metabolism
		GO:0034219 carbohydrate transmembrane transport	6.99 × 10^−18^	27	
		GO:0071702 organic substance transport	4.69 × 10^−12^	44	
		GO:0006810 transport	2.93 × 10^−8^	49	
		GO:0051234 establishment of localization	3.75 × 10^−8^	49	
		GO:0015749 monosaccharide transport	5.37 × 10^−8^	14	
		GO:0044765 single-organism transport	2.23 × 10^−7^	46	
		GO:0051179 localization	4.36 × 10^−7^	49	
		GO:0015768 maltose transport	4.45 × 10^−5^	6	
		GO:0009401phosphoenolpyruvate-dependent sugar phosphotransferase system	1.03 × 10^−4^	10	
		GO:0055085 transmembrane transport	1.20 × 10^−4^	36	
		GO:0015750 pentose transport	2.71 × 10^−4^	7	
		GO:0044724 single-organism carbohydrate catabolic process	6.79 × 10^−4^	21	
		GO:0015766 disaccharide transport	7.07 × 10^−4^	7	
		GO:0042956 maltodextrin transport	7.40 × 10^−4^	5	
		GO:0015772 oligosaccharide transport	1.61 × 10^−3^	7	
		GO:0016052 carbohydrate catabolic process	2.08 × 10^−3^	21	
		GO:0006004 fucose metabolic process	3.19 × 10^−3^	6	
		GO:0019521 D-gluconate metabolic process	7.58 × 10^−3^	6	
		GO:0044275 cellular carbohydrate catabolic process	8.56 × 10^−3^	11	
FG3	SG3 MG2 MG4	GO:0055080 cation homeostasis	7.34 × 10^−6^	8	Iron homeostasis pH homeostasis
		GO:0050801 ion homeostasis	9.10 × 10^−6^	8	
		GO:0048878 chemical homeostasis	1.37 × 10^−5^	8	
		GO:0042592 homeostatic process	3.33 × 10^−4^	8	
		GO:0055072 iron ion homeostasis	7.44 × 10^−3^	5	
		GO:0045852 pH elevation	9.50 × 10^−3^	3	
		GO:0051454 intracellular pH elevation	9.50 × 10^−3^	3	
FG4	SG4 MG1 MG5	No significant GO term			Glutamate biosynthesis (gltDF) Electron transport chain (cyoDE) Ferric enterobactin transport (fepA) Iron transport (fiu)
FG5	SG5 MG3	No significant GO term			Nitrite reductase (nirB) Acid resistance system (gadX)

**Table 3:** Detailed information on CRP-sFFLs and CRP-mFFLs. CRP-sFFLs and CRP-mFFLs are respectively composed of five and six groups involved in various types of FFLs (checked types). Five functional groups are listed using a functional group identifier. Each group is listed according to the expression pattern. The symbol V indicates that the type of FFL configuration contained in the Functional group.

Functional Group ID	Type	Group ID	Number of CRP-FFLs (Gene)	Coh1	Coh2	Coh3	Coh4	InCoh1	InCoh2	InCoh3	InCoh4
FG1	CRP-sFFLs	SG1	10 (10)	V				V			
FG2		SG2	78 (78)	V			V	V			V
FG3		SG3	10 (10)	V	V		V	V	V	V	
FG4		SG4	2 (2)					V			
FG5		SG5	1 (1)						V		
FG4	CRP-mFFLs	MG1	4 (2)		V	V					
FG3		MG2	15 (7)	V				V			V
FG5		MG3	2 (1)		V				V		
FG3		MG4	14 (6)		V				V		
FG4		MG5	6 (2)					V			V
FG2		MG6	60 (27)	V			V	V			V

## Supplementary Materials

The following are available online at http://www.mdpi.com/1422-0067/19/8/2335/s1.
